# A Prospective Cohort Study of Biomarkers in Squamous Cell Carcinoma of the Anal Canal (SCCAC) and their Influence on Treatment Outcomes

**DOI:** 10.7150/jca.57678

**Published:** 2021-10-16

**Authors:** Camila Motta Venchiarutti Moniz, Rachel Pimenta Riechelmann, Suilane Coelho Ribeiro Oliveira, Giovanni Mendonça Bariani, Thomas Giollo Rivelli, Cintia Ortega, Allan Andresson Lima Pereira, Sibele Inácio Meireles, Rejane Franco, Andre Chen, Renata Colombo Bonadio, Caio Nahas, Jorge Sabbaga, Renata Almeida Coudry, Maria Ignez Braghiroli, Paulo Marcelo Hoff

**Affiliations:** 1 Instituto do Cancer do Estado de Sao Paulo (ICESP), Hospital das Clinicas HCFMUSP, Faculdade de Medicina, Universidade de Sao Paulo, Sao Paulo, SP, BR.; 2 Instituto D'Or de Pesquisa e Ensino - IDOR, Sao Paulo, SP, BR.; 3 Clinical Oncology Department, AC Camargo Cancer Center, Sao Paulo, BR.; 4 Faculdade de Ciencias Médicas da Universidade Estadual do Piaui (UESPI), Piaui, BR.; 5 Hospital Sírio Libânes, Brasília, BR.; 6 Hospital Sírio Libânes, Sao Paulo, BR.; 7 Universidade Federal do Paraná - Hospital de Clínicas, Curitiba, PR, Brasil.; 8 UnitedHealth Group Brazil.

**Keywords:** Anal Carcinoma, Biomarkers, Ki-67, PD-L1, HPV, HIV

## Abstract

**Background:** Although Chemoradiation (CRT) is the curative treatment for SCCAC, many patients present primary resistance. Since it is a rare tumor, response predictors remain unknown.

**Methods:** We performed a prospective cohort study to evaluate biomarkers associated with CRT response, progression-free survival (PFS), and overall survival (OS). The primary endpoint was response at 6 months (m). Tumor DNA and HPV were analyzed by next-generation sequencing, while KI-67 and PD-L1 by immunohistochemistry in tumor tissue.

**Results:** Seventy-eight patients were recruited between October/2011 and December/2015, and 75 were response evaluable. The median age was 57 years, 65% (n=49) were stage III and 12% (n=9) were HIV positive (HIV+). At 6m, 62.7% (n=47) presented CR. On multivariate analyses, stage II patients were 4.7 more likely to achieve response than stage III (OR, 4.70; 95%CI, 1.36-16.30; p=0.015). HIV+ was associated with a worse response (OR, 5.72; 95%CI, 2.5-13.0; p<0.001). 5-year PFS and OS rates were 63.3% and 76.4%, respectively, with a median follow up of 66m. On multivariate analyses, older age (HR 1.06, p=0.022, 95%IC 1.01-1.11) and absence of CR at 6m (HR 3.36, p=0.007, 95%IC 1.39-8.09) were associated with inferior OS. The 5-year OS rate was 62.5% in HIV+ group compared to 78% among HIV- pts, although this difference was not statistically significant (p=0.4). *PIK3CA, MET* and* TP53* mutations, HPV, Ki-67 expression, and PD-L1 expression, were not associated with PFS and OS.

**Conclusions:** Clinical stage III and HIV+ were associated with worse response to CRT at 6m. The absence of CR was the main factor associated with poor 5-year OS.

## Introduction

CRT cures most patients with localized SCCAC. However, some tumors present primary resistance and 15% do not achieve complete clinical response (CR) at 26 weeks from CRT onset 1. Lack of response to CRT is a poor prognostic factor with a 5-year overall survival (OS) rate of 48% versus 87% among responders 1. Staging, on its own, is unable to explain all cases of uncontrolled disease, and only a few retrospective studies have been conducted to explore predictive CRT response biomarkers in SCCAC. In the absence of prospective studies in this scenario, stratification of recurrence risk and treatment intensification recommendations are lacking.

Immunosuppression and infection by the Human papillomavirus (HPV) play an important role in SCCAC. The integration of the HPV viral DNA in the host cells and oncoproteins E6/E7 production results in the inactivation of the tumor suppressor gene proteins pRb and p53 2,3, HPV also activates the PI3K/Akt/mTOR pathway, promoting cell proliferation 2. In terms of prognosis, HPV+, together with p16 positivity, has been associated with better local control at 5 years among SCCAC patients 4. A recent systematic review and meta-analyses and 17 retrospective cohort studies indicated that HPV +, and P16+, had improved OS 5. *TP53* mutations are described in SCCAC HPV- tumors and *PIK3CA* mutations were related to poor OS in patients who underwent salvage surgery post local relapse 6.

While HIV positivity is a known risk factor for developing SCCAC, randomized trials of SCCAC have consistently excluded HIV+ patients 7. Immune-mediated response against HPV and the tumor involves complex interactions among T cells, cytokines, and immune checkpoints. However, little is known about the intricacy of immune system operation during HPV, HIV, and tumor subsistence interaction 8,9. The literature on HIV+ SCCAC patient outcomes is scarce and studies about the predictive and prognostic impact of HIV status have reported conflicting results.

The presence of HPV integrated inside the nucleus of tumor cells makes the tumor more immunogenic, but cancer cells may express PD-L1 to neutralize the host immune response. The PD-1/PD-L1 checkpoint can be activated by the PI3K-AKT pathway, inducing upregulation of PD-L1 10. Phase II studies demonstrated immunotherapy activity in metastatic SCCAC, but the impact of PD-L1 expression in the primary tumor was unknown during radical treatment with curative intent 11. Another potential predictive biomarker in SCCAC is the Ki-67 index, as suggested by a systematic review, with a possible association of high Ki-67 with longer disease-free survival (DFS) 12.

Due to the rarity of this disease, the data available about predictive factors in SCCAC come from retrospective series, which are subject to numerous biases, including patients who are not treated with QT/RDT full dose and analyses of tumor samples from different sites and treatment stages.

We aimed at detecting, in a prospective trial, if clinical factors or tumor biomarkers analyzed in the primary tumor before starting treatment (HIV, HPV, PD-L1, Ki-67, and DNA tumor mutations) were associated with CRT efficacy.

## Materials and Methods

Consecutive patients with localized SCCAC at the “Instituto do Câncer do Estado de São Paulo'' were invited to participate in this study, from October/2011 to December/2015. Inclusion criteria were histological diagnosis of invasive SCCAC, stage T2-4 N0 M0 or T1-4 N1-3 M0 according to the AJCC VII 13, ≥ 18 years old, and be candidates for complete treatment with curative intent. Standard treatment includes radiotherapy 45-54Gy plus a single dose of mitomycin 15 mg/m^2^ on D1 and 5-fluorouracil continuous infusion 1000 mg/m^2^/day on D1-4 and D29-32. In addition, Capecitabine 825 mg/m^2^ orally BID could be used during radiotherapy as a substitute for 5-FU. The sample was estimated as 70 patients based on convenience and feasibility.

HIV tests and tumor samples were obtained before CRT started. Clinical examination and imaging with Response Criteria in Solid Tumors (RECIST) v1.1 14 were performed at 6-8 weeks and at six months (+/-15 days) after CRT to assess response. Chest and abdominal tomography (CT) plus pelvic CT or magnetic resonance image (MRI) were performed during initial staging and follow-up. After six months, patients were followed every three months during the first two years and every six months from the third to fifth. Imaging was performed annually from the first to the fifth year. During CRT, toxicities were graded using the Common Terminology Criteria for Adverse Events, v3.0 15. Laboratory tests were performed before D1 and D29 of CRT. We also collected information on age, sex, ECOG, and CRT dose-intensity.

Tumor DNA was assessed for integrity by real-time polymerase chain reaction (PCR) using specific primers included in TruSight Tumor 26-Illumina® kit, that sequence specific exons in 26 genes:* AKT1, ALK, APC, BRAF, CDH1, CTNNB1, EGFR, ERBB2, FBXW7, FGFR2, FOXL2, GNAS, GNAQ, KIT, KRAS, MAP2K1, MET, MSH6, NRAS, PDGFRA, PIK3CA, PTEN, SMAD4, SCR, STK11,* and* TP53.* Sample preparation followed the manufacturer's guidelines (www.illumina.com). At least 1000× coverage of each allele and variant frequency >5% were considered for analysis. The identification and classification of variants was performed by Variant Studio Software plus manual online database research at cBioPortal (http://www.cbioportal.org/), Clinvar (https://www.ncbi.nlm.nih.gov/clinvar), Varsome (https://varsome.com) and The Clinical Knowledgebase (https://ckb.jax.org/).

HPV screening was performed by PapilloCheck® kit (Greiner Bio-One GmbH) that identifies HPV 6, 11, 16, 18, 31, 33, 35, 39, 40, 42, 44, 55, 45, 51, 52, 53, 56, 58, 59, 66, 68, 70, 73, 82. When sample quality was insufficient for PapilloCheck®, hybridization *in situ* using the INFORM HPV III Family 16 and INFORM HPV II Family 6 (Ventana®) were performed to identify HPV 6,11, 16, 18, 31, 33, 35, 39, 45, 51, 52, 56, 58 and 66. Immunohistochemical reactions were performed with mouse anti-human Ki-67 clone MIB-1-DAKO® (1:200) and anti-PD-L1 XP E1L3N clone Cell Signaling®(1:100), according to the manufacturer's guidelines.

The primary study objective was 6 months response rate. Association between biomarkers (HPV status, ki67, PD-L1, mutations) and clinical variables (stage, age, HIV status, and clinically relevant treatment interruption, defined as a pause of more than 7 days in radiotherapy and/or not completing full chemotherapy plan) and response rate (CR and CR plus PR combined) at six months was assessed by univariable and multivariate logistic regression. Two-sided p values <0.05 were considered statistically significant in multivariate logistic regression. Comparisons of categorical variables between groups were performed by Fisher's exact test.

Secondary objectives were OS and PFS. OS was calculated from the date of C1D1 treatment until the date of death from any cause. PFS was considered the time from the date of C1D1 treatment to clinical progression, radiological progression, or death from any cause. Patients without the events were censored at the date of the last follow-up. The Kaplan-Meier method was used to estimate survival functions, and survival curves were compared using the log-rank test. Factors associated with OS were evaluated with univariable and multivariate Cox regression. The potential prognostic factors evaluated were age, stage, treatment interruption, HIV status (positive vs. negative), smoking status (yes vs. no), ECOG (2/3 vs. 0/1), HPV status (yes vs. no), CR at 6m (no vs. yes), KI-67 (≥50% vs. <50%), PD-L1 > 1% (yes vs. no), codon 72 polymorphism in *TP53* (presence vs. absence), *MET* mutations (presence vs. absence),* PIK3CA* mutations (presence vs. absence). In the Cox multivariate analysis, p values < 0.05 were considered statistically significant. Stata Software, version 15 (StataCorp, Texas, USA), was used for statistical analyses. Our local Ethics Committee approved the study and all eligible patients signed an informed consent form.

## Results

From October/2011 to December/2015, we recruited 78 patients. Two patients were excluded due to metastatic disease on initial staging in image review and one withdrew from the study. Seventy-five patients were evaluable for response and 68 had tumor tissues available for biomarker evaluation. Four patients were defined as having CR at 6 months based only on clinical examination because three did not have measurable disease at baseline and one did not perform pelvic imaging. Clinical examination included local evaluation anoscopy and digital rectal exam in all patients. All these 4 patients had sustained CR for more than 2 years. The median age was 57 years, 54 patients (72%) were female, 49 (65%) presented stage III disease, nine (12%) were HIV+, and the majority (93.3%) was ECOG 0/1 (Table 1). All pts HIV + included are male, and the majority had stage III (Table 2).

At 6-8 weeks after treatment, 41 patients (54.6%) attained CR, 28 (37.3%) presented partial response (PR), two (2.7%) had stable disease, and three (4%) experienced progression (PD) as best response. At six months after CRT, 47 (62.7%) patients achieved CR, 18 (24%) PR, and 10 (13.3%) PD only. All patients who hadn't presented either CR or PR at 6-8 weeks, developed PD at six months.

At the six months evaluation, the outcomes among the HIV+ (n=9) and HIV- (n=66) groups were, respectively: CR 33% (n=3) versus 66% (n=44) (p=0.07), PR 33% (N=3) versus 22.7% (n=15) (p=0.44), and PD 33% (n=3) versus 10.6% (n=7) (p=0.09). All HIV patients with PD received full-planed CRT. The outcomes among the stage II (n=26) and III (n=49) were, respectively: CR 84.6% (n=22) versus 51% (n=25) (p=0.005), PR 11.6% (N=3) versus 30.6% (n=15) (p= 0.08), and PD 3.8% (n=1) versus 18.4% (n=9) (p=0.15).

The most common grade 3/4 toxicities were lymphopenia in 62 (82.6%) and radiodermatitis in 45 (60%) patients. Seventy percent (N=53) had clinically relevant treatment interruption, mostly due to radiodermatitis. Two patients had febrile neutropenia and no treatment-related deaths occurred.

HPV was evaluated in 67 patients and found in 47 (70.1%), and 45 patients (60%) presented high-risk HPV, considering *in situ* hybridization and PapilloCheck® evaluation.

It was possible to perform the PapilloCheck® test in 39 patients. Twenty-five had HPV positive samples being the most frequent type was the HPV 16 in 20 patients, followed by HPV 35 in three patients.

PD-L1 was tested in 61 patients, with 10 (16.4%) being positive (>1% positive expression). Ki-67 was performed in 65, and the median value was 50% (range 1-90%). HIV+ patients exhibited a higher Ki-67 (median 80%) than HIV- (median 50%).

On univariable analysis of CR at six months, the stage was associated with CR (OR, 5.28; 95% CI, 1.58-17.59; p=0.007). On multivariate analysis, stage II patients were 4.7 times more likely to attain CR than those with stage III (OR, 4.70; 95% CI, 1.36-16.30; p=0.015). HIV-positive patients had a trend towards lower CR rate (OR, 2.53 95% CI, 0.9-7.1; p=0.079). Considering CR plus PR in a separate multivariate model, HIV+ was significantly associated with lower rates of response (CR plus PR) at six months (OR, 5.72; 95% CI, 2.5-13.0; p<0.001). Results of the logistic regression of factors associated with complete response at 6 months are shown in Table 4.

NGS was performed in samples of 27 patients, 25 of whom had a response evaluation. At least one mutation was found in 17 (17/25, 68%) (Table 3). The most commonly mutated genes were *PIK3CA* and* MET* in six patients each. The mutation described in MET was initially not found in the genomic databases. Later, during the evolution of the cohort, it was described as potentially benign. Due to poor information available in the literature, we chose to keep the *MET* mutation in the study analyses. Six-month CR rates were similar according to *MET* mutation status (50% in *MET*-mutated vs. 47.3% in* MET*-wild type, p=1.0); or *PIK3CA* mutation status (33.3% in *PIK3CA*-mutated vs. 47.3% in *PIK3CA*-wild type, p=0.6). The c.215C>G *TP53* polymorphism was found in 72% (18 of the 25 patients who were tested) and wasn't associated with CR either (44% in those with *TP53* codon 72 polymorphism vs. 57% in those without *TP53* codon 72 polymorphism, p=0.6). Among patients who did not carry polymorphisms (n=7) none presented PD at 6 months, while PD at 6 months occurred in 33% (n=6) of those with the polymorphism.

**Figure 1:**
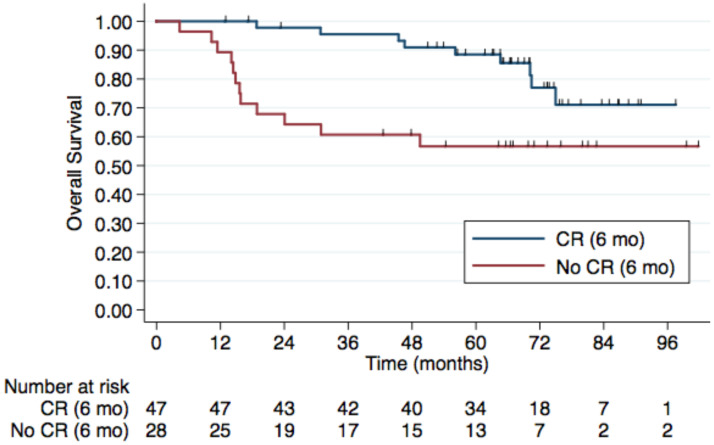
The 5-year OS rates were 88.5% in CR group vs. 56.6% in non-CR group (HR 3.36, 95% CI, 1.39-8.09; p=0.007).

After a median 66-month follow-up of the 75 patients, 21 deaths occurred. In multivariate analyses, older age, and absence of complete response at 6 months were associated with inferior PFS and OS. The 5-year OS rates were 88.5% in CR group vs. 56.6% in non-CR group (HR 3.36, 95% CI, 1.39-8.09; p=0.007) (Figure 1). Univariable and multivariate analyses of factors associated with OS are shown in Table 5.

**Figure 2:**
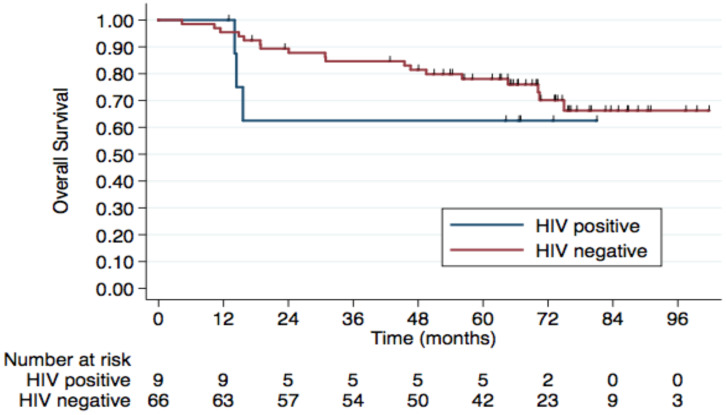
** 5-year Overall Survival by HIV Status.** The 5-year OS rates were 78% in the HIV- group vs. 62.5% in the HIV+ group, although this difference was not statistically significant (p=0.4).

**Figure 3:**
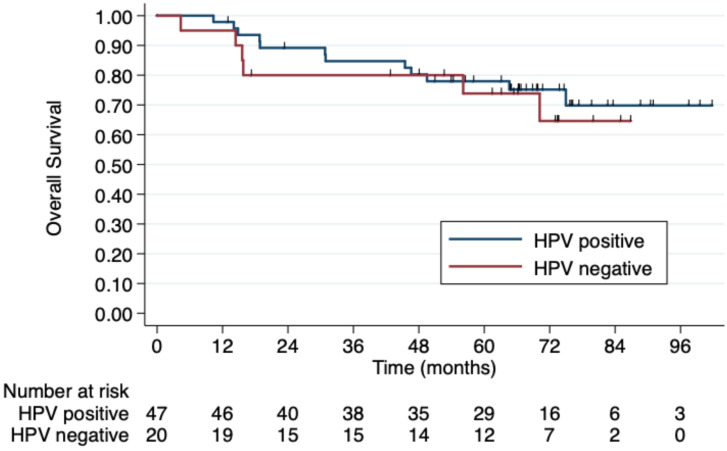
** 5-year overall survival by HPV status.** No difference in OS was observed according to HPV status.

**Figure 4:**
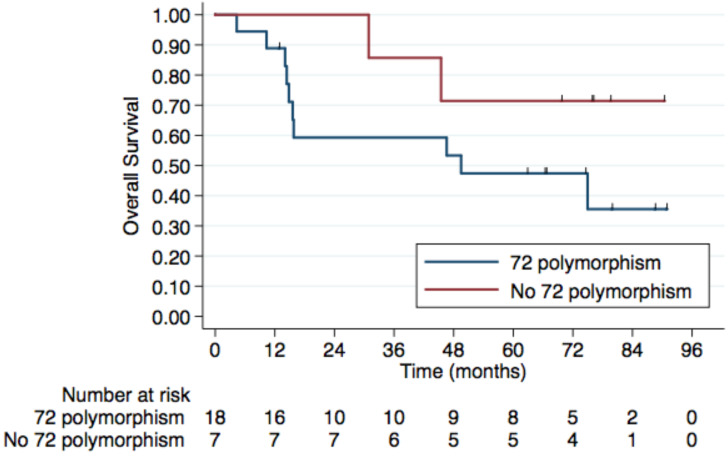
** 5-year Overall Survival by *TP53* codon 72 polymorphism.** Patients without TP53 codon 72 polymorphism have a 5-year OS of 71.4% compared to. 47.4% in those with a TP53 codon 72 polymorphism, although this difference was not statistically significant (p=0.181).

Regarding HIV status, the 5-year PFS rates were 64.7% in HIV-negative patients and 53.3% in those HIV-positive. The 5-year OS rates were 78% and 62.5%, respectively, although this difference was not statistically significant (p=0.4) (Figure 2). No difference was observed according to HPV status either (Figure 3). Patients without TP53 codon 72 polymorphism presented 5-year PFS 71.4% vs. 37% and 5-year OS 71.4 vs. 47.4% (p=0.181) (Figure 4).

## Discussion

Our prospective cohort study showed that most patients had HPV+ and stage III tumors, and 12% were HIV positive. Our CR rate at 6 months was 62.7% lower than observed in the mitomycin arm in ACT II trial at 26 weeks (90.5%). In ACT II, around 50% of patients had T1/T2 disease and 63% node negative disease 7. Our population had a high number of stage III bulky disease. Our stage II patients had a significantly better response to treatment, with 84.6% CR at six months.

Retrospective series showed heterogeneous results, some with complete response rates similar to historical controls in HIV negative (HIV-) 16 and others with lower OS in the HIV+ group 17. Our population had 12% HIV positive, and this study demonstrates that HIV is an important biomarker, influencing response to CRT. Only one-third of HIV+ patients achieved CR at six months, contrasting with 66.7% of the HIV- group. Also, in the multivariate analysis, HIV- patients were 5.7 times more likely to achieve CR or PR than HIV+. Although HIV status was not associated with survival, CR at 6 months was associated with better PFS and OS in a multivariate model.

Our tumor samples had 70.1% HPV+, in agreement with the literature that showed HPV in 78% of SCCAC, the majority HPV16 18. In our study, HPV did not significantly influence CR, PFS and OS when prospectively evaluated together with other relevant factors. One possibility is that retrospective cohorts evaluated in the available metanalyses had selection biases and confounders that influenced their results 5.

Ki-67 protein is a nuclear antigen found in proliferating cells 12. In Ajani 19 study, Ki-67 had significant association with DFS and response. In our study, the ki-67 index did not influence CR at six months (OR, 0.9; 95% CI, 0.9-1; p=0.18). In previous studies, the HIV status was not evaluated systematically and may have been an occult information bias. In our setting, Ki-67 was not a useful marker to predict response, PFS or OS.

In order to evade antitumor immunity T cell mediated pathway, some tumor cells produce the programmed cell death-ligand 1 (PD-L1) and reduce T cells activation through bonding at a down regulated receptor, PD-1, on surface T cells 20-22. A study suggested that 50% of SCCAC had PD-L1 expression, but 31% of samples were from recurrent tumors 8. Immunotherapeutic drugs emerged as a promising treatment in refractory metastatic SCCAC, showing an objective response in 24% 22. In a retrospective analysis of 41 tumor samples, PD-L1 positive was associated with poor recurrence rates 23. In our study, 16.4% of tumor samples had PD-L1 positive expression and did not influence CR at six months. All our samples were obtained from the primary tumors and prior to CRT, which may explain our different results.

PIK3CA is the most frequent tumor alteration in SCCAC, reported in up to 40% of patients 24. While we did not observe a difference in CR according to *PIK3CA* mutations, our sample was small and not powered to evaluate differences in response rates. The phase I study with Taselisib, a PI3K pathway inhibitor, demonstrated activity in H1047 mutated tumors 25. In our study, six patients were carriers of *PIK3CA* mutation, two in H1047 (Table 4), revealing the potential use of PI3K inhibitors in SCCAC. *MET* pathway crosstalk with PIK3/AKT signaling and *MET* mutations may carry the cell to a metastatic phenotype. In our study, *MET* mutations were frequent (24%) but did not predict CR. Smaglo 26 observed only one mutation in 57 samples. MET-targeted therapies, including crizotinib, cabozantinib, capmatinib, tepotinib and glesatinib are being investigated in patients with *MET* mutations 27.

A retrospective study performed in 148 patients observed *TP53* mutations in 4.7%, the majority among HPV negative (HPV-) samples. In our study, three patients had *TP53* mutation, only one HPV- (Table 3). The E6 HPV oncoprotein interacts with p53 and causes its degradation, and the presence of the germline polymorphism *TP53* c.215C>G may increase it 28-30. Homozygote's individuals showed a 7-fold increased risk of HPV related cancers 31. We found *TP53* c.215C>G in 72% of tumor samples, and it was not associated with CR at 6 months (44% vs. 57%, p=0.6). However, an intriguing fact was that at six months, no progression was observed among patients without polymorphism. At 5-year follow-up, patients without *TP53* c.215C>G presented better PFS and OS, but this difference was not statistically significant in this cohort.

We observed low mutation rates in other relevant genes (Table 3) described in solid tumors, in agreement with the literature: *BRAF* 0%, *KRAS* in 0-4%, *EGFR* 0-3% 32, 33.

Our study had limitations, it was a single-center study and the quality/size of the tumor samples permitted molecular studies in only 38%. On the other hand, all patients were systematically treated and followed. A single experienced radiologist and pathologist reviewed, respectively, all the images and biopsy. To our knowledge, this is the first study to analyze biomarkers prospectively in SCCAC.

## Conclusion

This cohort study suggests that HIV- patients had 5.7 times more chance of achieving CR or PR than HIV+ at six months post CRT. Clinical stage and HIV+ were associated with worse response to CRT at 6m.

The absence of complete response at 6 months was the main factor associated with poor 5-year OS, and new strategies of follow-up and complementary approaches should be studied in these patients to ensure curative treatment success.

In this study, HPV, *PIK3CA* or* MET* mutations, Ki-67, and PD-L1 expression did not influence response rate, PFS, and OS. The TP53 c.215C>G polymorphism may potentially predict PFS and OS curves and need to be evaluated in larger prospective.

## Implications for practice

In this prospective study, HIV- patients had 5.7 times more chance of achieving CR or PR than HIV+ at six months post CRT. Considering 5-year OS, the absence of complete response at six months was the main factor associated with a poor prognosis.

Special attention is required for the patient's HIV+ or without a complete response at six months post CRT. It's time to develop and study new strategies for follow-up and complementary approaches in these populations.

## Figures and Tables

**Table 1:** Population Characteristics.

Baseline Characteristics (n=75)		
Characteristic	n	(%)
**Sex**		
Female	54	72
Male	21	28
**HIV Status**		
Positive	9	12
Negative	66	88
**ECOG**		
0	34	45.3
1	36	48
2	4	5.3
3	1	1.4
**Stage**		
II	26	34.7
IIIA	19	25.3
IIIB	30	40
**Primary Tumor (T)**		
T1	1	1.4
T2	31	41.3
T3	18	24
T4	25	33.3
**Regional Lymph Nodes (N)**		
N0	35	46.7
N1	15	20
N2	18	24
N3	7	9.3
**Median Age - years (range from)**		
HIV positive	50 (42; 60)	
HIV negative	56 (38; 79)	
All patients	57 (38; 79)	

**Table 2:** Study Population Characteristics according to HIV Status

Study population characteristics according to HIV status			
	HIV + (n=9)	HIV - (n=66)	*p* value
**Sex**			<0.0001
Female	0	54	
Male	9	12	
**ECOG**			1
ECOG 0/1	9	61	
ECOG 2/3	0	5	
**Stage**			1
II	3	23	
III	6	43	
**Primary Tumor (T)**			0.15
T1/T2	6	26	
T3/T4	3	40	
**Regional Lymph Nodes (N)**			1
Negative	4	31	
Positive	5	35	
**Age,** mean (± SD**)	58 (±9.9)	50 (± 5.6)	0.02

*P value were based in two-tailed Fisher's exact test for categorical variables, and independent t-test for continuous variables. ** SD: Standard Deviation.

**Table 3:** 27 samples were submitted to molecular analyses and 25 patients had response evaluation by RECIST after QRT treatment. HPV can be identified by type on samples suitable to the PapilloCheck® —patients who performed only *in situ* hybridization show positive or negative results for the presence of HPV.

Mutations in SCCAC (N=27)									
Gene	Exon	Variant	Nucleotide Change	Protein predictive change	Mutation type	Classification	HIV	HPV	Sample
*APC*	15	C>C/T	c.3985C>T	p.His1329Tyr	Missense	Unknown Significance	-	+HPV16	48
*BRAF*	11	C>C/T	c.1379G>A	p.Gly460Glu	Missense	Unknown Significance	-	+	42
*CDH1*	12	G>G/A	c.1849G>A	p.Ala617Thr	Missense	Benign	+	+HPV16	30
*FBXW7*	10	C>C/T	c.1420G>A	p.Val474Ile	Missense	Unknown Significance	-	+HPV16	38
*FBXW7*	10	C>C/T	c.1550G>A	p.Gly517Glu	Missense	Unknown Significance	-	+HPV16	05
*FBXW7*	12	G>G/A	c.1972C>T	p.Arg658Ter	Stop Gain	Presumed Pathogenic	-	+HPV35	31
*KRAS*	5	C>C/T	c.564G>A	p.Met188Ile	Missense	Unknown Significance	-	+HPV16	48
*MET*	2	C>C/T	c.1075C>T	p.Arg359Ter	Stop Gain	Unknown Significance	-	+	24
*MET*	2	C>C/T	c.818C>T	p.Thr273Ile	Missense	Unknown Significance	+	-	32
*MET*	2	G>G/A	c.457G>A	p.Asp153Asn	Missense	Unknown Significance	+	+HPV16	36
*MET*	2	T>T/A	c.607T>A	p.Ser203Thr	Missense	Likely Benign	-	+HPV16	48
*MET*	21	TC>TC/T	c.4058delC	p.Arg1354GlyfsTer17	Frameshift	Unknown Significance	-	+HPV16	51
*MET*	2	G>G/T	c.504G>T	p.Glu168Asp	Missense	Likely Benign	+	-	76
*NRAS*	4	G>G/A	c.304C>T	p.Arg102Ter	Stop Gain	Unknown Significance	-	+HPV16	16
*PDGFRA*	18	G>G/A	c.2458G>A	p.Ala820Thr	Missense	Unknown Significance	-	+HPV16	36
*PIK3CA*	9	G>G/A	c.1633G>A	p.Glu545Lys	Missense	Pathogenic	-	+HPV16	01
*PIK3CA*	21	A>A/T	c.3140A>T	p.His1047Leu	Missense	Pathogenic	+	+HPV16	36
*PIK3CA*	21	A>A/G	c.3140A>G	p.His1047Arg	Missense	pathogenic	-	+HPV16	39
*PIK3CA*	9	G>G/A	c.1645G>A	p.Asp549Asn	Missense	Pathogenic	-	+	57
*PIK3CA*	9	G>G/A	c.1645G>A	p.Asp549Asn	Missense	Pathogenic	-	+HPV16	31
*PIK3CA*	9	C>C/A	c.1636C>A	p.Gln546Lys	Missense	Pathogenic	-	+HPV35	74
*PTEN*	7	C>C/T	c.733C>T	p.Gln245Ter	Stop Gain	Pathogenic	+	+HPV16	30
*TP53*	9	TC>TC/T	c.970delG	p.Asp324MetfsTer21	Frameshift	Pathogenic	-	+HPV16	48
*TP53*	8	G>G/A	c.817C>T	p.Arg273Cys	Missense	Pathogenic	+	+HPV 06	25
TP53*	7	C>C/T	c.733G>A	p.Gly245Ser	Missense	Pathogenic	-	-	78

* Mutation in a patient not evaluable for response.

**Table 4:** In logistic regression - multivariate analysis, HIV positive is significantly associated with less chance of response (CR or PR) at 6 months post definitive chemoradiation.

Factors associated with 6-month response rate by Logistic regression						
	Univariable Analysis			Multivariate Analysis		
	OR	p	95% CI	OR	p	95% CI
Age (continuous variable)	1.02	0.57	0.95-1.09			
Stage (III vs. II)	5.62	0.11	0.67-47.1	6.517	0.080	0.80-53
HIV (positive vs negative)	4.2	0.07	0.85-20.7	**5.720**	**<0.001**	**2.51-13.0**
HPV (positive vs negative)	1.48	0.61	0.31-6.9			
Ki-67 (≥ 50% vs < 50%)	0.98	0.4	0.95-1.07			
PD-L1 > 1% (yes vs no)	1.02	0.98	0.10-9.83			
Treatment Interruption (yes vs no)	0.96	0.9	0.22-4.12			

**Table 5:** In multivariate analyses, older age and absence of complete response at 6 months were associated with inferior OS.

Factors associated with overall survival (Cox regression)						
	Univariable Analysis			Multivariate Analysis		
	HR	p	95% CI	HR	p	95% CI
Age (continuous variable)	1.04	0.046	1.00-1.09	**1.06**	**0.022**	**1.01-1.11**
Stage (III vs. II)	0.93	0.883	0.38-2.25			
HIV (positive vs negative)	1.69	0.400	0.49-5.76			
Smoking (yes vs no)	1.45	0.482	0.51-4.16			
ECOG (2-3 vs 0-1)	3.09	0.072	0.90-10.58			
HPV (positive vs negative)	0.77	0.603	0.28-2.05			
Ki-67 (≥ 50% vs < 50%)	1.10	0.832	0.42-2.85			
PD-L1 > 1% (yes vs no)	0.38	0.351	0.05-2.88			
*MET* mutation (yes vs no)	2.08	0.234	0.62-7.01			
*PIK3CA* mutation (yes vs no)	1.98	0.267	0.59-6.62			
c.215C>G *TP53* polymorphism (yes vs no)	2.83	0.181	0.61-13.02			
Absence of Complete Response at 6m (yes vs no)	2.95	0.014	1.24-7.02	**3.36**	**0.007**	**1.39-8.09**
Treatment Interruption (yes vs no)	0.91	0.843	0.36-2.26			

## Abbreviations

CR: complete response
CRT: Chemoradiotherapy
HIV: human immunodeficiency virus
HPV: human papillomavirus
m: months
PD-L1: programmed cell death-ligand 1
PR: partial response
SCCAC: Squamous Cell Carcinoma of the Anal Canal
OS: Overall survival
PFS: progression free survival
